# Theoretical Design of All-Optical Universal Logic via Multistage FRET-STED Energy Cascades in Fluorescent Protein Nanoclusters

**DOI:** 10.1021/acsomega.6c03748

**Published:** 2026-07-06

**Authors:** Balázs Rakos

**Affiliations:** Department of Automation and Applied Informatics, 61810 Budapest University of Technology and Economics, Budapest H-1117, Hungary

## Abstract

The scaling limits of conventional electronics necessitate a shift toward alternative physical computing substrates. We present a universal logic design based on a novel materials platform employing FRET-coupled Fluorescent Protein (FP) NOR gates. By leveraging the nonlinear dynamics of Stimulated Emission Depletion (STED) to bypass slow protein backbone rearrangements, this system enables all-optical switching at speeds ranging from 1 GHz to potentially even 1 THz. We developed a dynamic multistage model of a three-protein FRET-STED cascade to evaluate performance. While a “lifetime bottleneck” initially limits standard operation, we demonstrate that engineering the plasmonic environment via Nanometal Surface Energy Transfer (NSET) enables 1 GHz operation with current protein variants. Analysis of fundamental electronic transitions—specifically vibrational relaxation—confirms a theoretical bandwidth of ∼3.15 THz for optimized synthetic chromoproteins. This work establishes a robust foundation for high-speed, nanoscale, all-optical computing using DNA origami as a molecular breadboard for precise sub-10 nm positioning.

## Graphical abstract

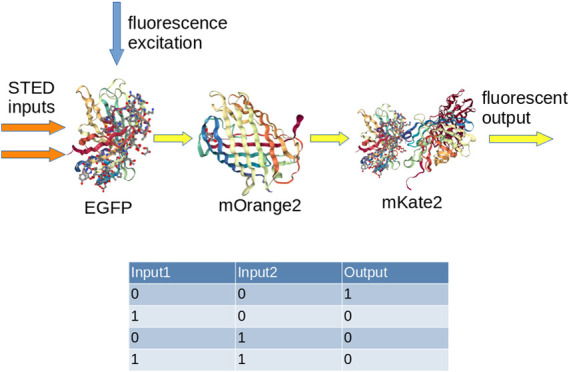

## Introduction

The relentless pursuit of computational power, governed for decades by Moore’s Law, is currently confronting a fundamental “scaling crisis” as silicon-based semiconductor technology approaches its physical miniaturization limits.^1^ Beyond the lithographic constraints, the management of power density and heat dissipation in sub-10 nm nodes presents a significant barrier to further performance gains.^2^ This “performance chasm” has catalyzed a paradigm shift toward molecular logic, where computational intelligence is embedded directly into matter at the nanometer scale.^1,3^ Since the seminal work by de Silva in 1993, the field of molecular logic has expanded into a robust discipline, converting continuous chemical and physical inputs into discrete binary digital states through defined thresholding mechanisms.^4,5^

While nucleic acid–based logic cascades have demonstrated remarkable scalability through strand displacement, they are inherently limited by diffusion-controlled kinetics, often operating on time scales of hours to days.^1,2^ Proteins offer a superior alternative due to their structural diversity, self-assembly capabilities, and faster allosteric transitions.^6^ Recent advancements have yielded autonomous single-protein devices, such as the two-input logic OR gate; however, even these state-of-the-art allosteric switches remain constrained to the minute-range for logic completion.^1,7^

A significant bottleneck in current protein-based logic, particularly those employing reversibly switchable fluorescent proteins (RSFPs), is the dependence on protein backbone rearrangements. For instance, the positive-switching RSFP Kohinoor exhibits a critical on-like intermediate state (*C*_Int_) with a lifetime of ∼1 ms and a triplet state relaxation time (τ_1_) of 2.46 ± 0.32 ms.^8^ These millisecond-to-microsecond transitions are orders of magnitude slower than the fundamental electronic limits of the chromophore. In contrast, the initial photoisomerization event and intramolecular vibrational energy redistribution (IVR) occur on a sub-100 fs time scale.^9,10^ Specifically, the structural transition for isomerization in fluorescent proteins is completed within ∼180 fs, corresponding to a theoretical experimental bandwidth of 3.15 THz.^9^

In this work, we propose a novel materials platform that bypasses slow conformational switching by utilizing Stimulated Emission Depletion (STED) as the primary logic mechanism. By shifting the switching logic from backbone-dependent isomerization to near-instantaneous electronic depletion, we design a universal NOR gate based on multistage FRET-STED energy cascades in fluorescent protein nanoclusters. We develop a coupled dynamic model incorporating competing rates of excitation, energy transfer, and depletion, verifying the feasibility of 1 GHz to potentially 1 THz operation. Finally, we outline a verification roadmap utilizing DNA origami as a ”molecular breadboard” for sub-10 nm positioning and intensity-based STED-FRET microscopy to resolve logic interactions at the single-molecule level.^11,12^

## Concept

We propose a concept for realizing binary, universal logic circuits using only fluorescent proteins or, more generally, fluorescent molecules. The central component of this arrangement is a NOR gate, implemented using a single molecule or a cluster of molecules of the same type.

The gate operates as follows: The fluorescent protein or aggregate is continuously irradiated by its excitation radiation, causing it to emit fluorescent light (output logic “1”). Photon beams that cause stimulated emission depletion (STED) in the molecule serve as the input signals. If either of the input signals is present (input logic “1”), STED occurs, causing the fluorescence to cease (output logic “0”).

Since coupling between logic gates is essential in a logic circuit, a minimum of three types of fluorescent proteins are necessary for the system (see Figure 1). One molecule or cluster realizes the NOR gate (*protein*_1_), while the other two proteins or protein clusters (*protein*_2_ and *protein*_3_) are required for coupling the output signal to the next NOR gate in the circuit.

**Figure 1:**
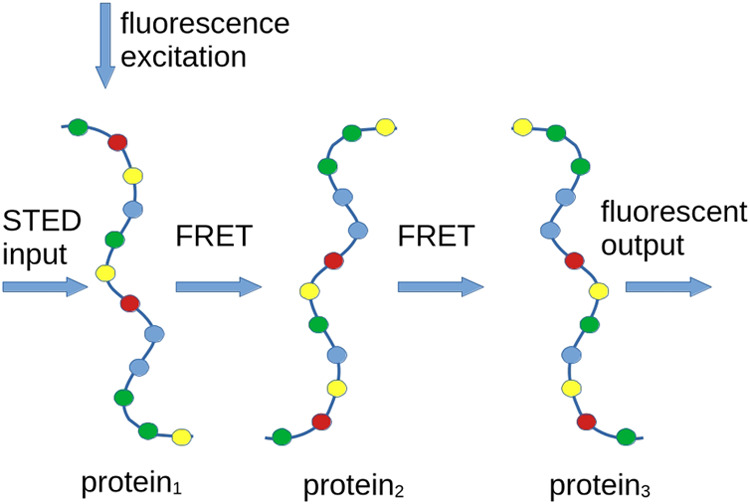
Fluorescent protein-based NOR gate concept.

At the nanoscale dimensions envisioned (intercluster spacing ≈ 5 nm), the dominant energy transfer mechanism between these clusters is Förster Resonance Energy Transfer (FRET), a nonradiative dipole–dipole interaction, rather than traditional far-field photon coupling.

This arrangement operates as follows: the excited-state energy of *protein*_1_ is transferred to *protein*_2_ via FRET, which in turn excites *protein*_3_. The output from *protein*_3_ is used for the depopulation of *protein*_1_ of the next NOR gate. Note that this stable cascaded operation requires at least three distinct spectral stages. A two-cluster arrangement would be physically untenable because the energy transfer or emission from the coupling stage would interfere with the donor stage of the same gate, creating an uncontrolled feedback loop. By utilizing a three-protein FRET chain, we ensure unidirectional signal propagation and provide sufficient spectral separation to maintain logic integrity.

Since NOR gates are universal logic gates, this method is suitable for realizing universal binary logic circuits, provided that molecules with suitable properties are used. However, this method naturally lacks signal amplification. To prevent signal loss throughout the system, amplifying elements must be introduced periodically. We propose that photoswitchable fluorescent proteins, utilized in a separate stage from the NOR gate, are excellent candidates for this purpose, where the output intensity is independent of the input signal.^13^

## Model

To rigorously evaluate the feasibility and transient behavior of the proposed FRET-coupled logic architecture, we developed a dynamic multistage model based on coupled rate equations. This framework incorporates the stochastic nature of molecular transitions and the competing rates of constant excitation, stimulated emission depletion (STED), and nonradiative energy transfer. Furthermore, to ensure ”physical reality,” we integrated substrate-induced quenching effects via nanometal surface energy transfer (NSET) and established a localized thermal energy budget to address the risk of photothermal denaturation during high-intensity operation. This multiphysics approach enables a detailed analysis of the proposed architecture.

### Equations

A second-order bandpass filter characteristic^13^ is utilized to provide the frequency-selectivity for fluorescence excitation and STED. The transfer function of the filter is defined as

1 $$H\left(\omega \right)=\frac{\frac{\omega_{0}}{Q}i\omega }{{\left(i\omega \right)}^{2}+\frac{\omega_{0}}{Q}i\omega +\omega_{0}^{2}}$$

where ω is the angular frequency, *Q* is the quality factor, *i* is the imaginary unit, and ω_0_ is the resonant angular frequency for either the fluorescence excitation or STED radiation.

Applying eq 1, the intensity of the radiation affecting the molecule, *I*_*i*_(*t*), is expressed by

2 $$I_{i}\left(t\right)=\int_{0}^{\infty }\left|H\left(\omega \right)\right|I_{i0}\left(\omega ,t\right)d\omega$$

where *I*_*i*0_(ω, *t*) is the intensity of the incident radiation. If the radiation possesses a discrete spectrum, this equation simplifies to

3 $$I_{i}\left(t\right)=\left|H\left(\omega_{1}\right)\right|I_{i01}\left(\omega_{1},t\right)+\left|H\left(\omega_{2}\right)\right|I_{i02}\left(\omega_{2},t\right)+...$$

To accurately model high-speed transients, the system is described by the instantaneous excited state population, N*­(*t*). The power of the fluorescent output radiation is determined by the rate of spontaneous emission from this population

4 $$P_{f}\left(t\right)=\frac{N^{*}\left(t\right)\cdot \phi_{f,\mathrm{eff}}}{\tau_{f,\mathrm{eff}}}\cdot E_{\mathrm{photon}}$$

where ϕ_*f*,eff_ is the effective quantum yield, τ_*f*,eff_ is the effective fluorescence lifetime, and *E*_photon_ is the energy of the emitted photon. At the single-molecule level, N*­(*t*) represents the probability of the molecule being in the excited state. In the presence of STED radiation, the excited state population is governed by a dynamic rate equation that accounts for the competition between constant excitation and stimulated depletion

5 $$\frac{dN^{*}\left(t\right)}{dt}=\sigma_{\mathrm{exc}}I_{e}\left(t\right)\left(N_{total}-N^{*}\left(t\right)\right)-\sigma_{sted}I_{s}\left(t\right)N^{*}\left(t\right)-\frac{N^{*}\left(t\right)}{\tau_{f,\mathrm{eff}}}$$

where σ_exc_ and σ_sted_ represent the absorption and STED cross sections, and *I*_*e*_(*t*) and *I*_*s*_(*t*) are the instantaneous intensities of the excitation and STED radiation, respectively.

### Nonradiative Energy Transfer (FRET^14–17^ ) Coupling

In a cascaded logic sequence, the relaxation of a donor molecule (*D*) nonradiatively excites an acceptor molecule (*A*). The efficiency of this transfer, *E*, depends on the intermolecular distance *d* and the Förster distance *R*_0_

6 $$E=\frac{1}{1+{\left(d/R_{0}\right)}^{6}}$$

The rate of energy transfer, *k*_ET_, is defined by the donor’s excited state lifetime τ_*D*_ and the coupling efficiency

7 $$k_{\mathrm{ET}}=\frac{1}{\tau_{D}}{\left(\frac{R_{0}}{d}\right)}^{6}$$

The critical Förster distance, *R*_0_, at which the energy transfer efficiency is 50%, is defined by the spectral properties of the donor–acceptor pair and their transition dipole orientations

8 $$R_{0}^{6}=\frac{9000\left(\ln10\right)\kappa^{2}\Phi_{D}}{128\pi^{5}N_{A}n^{4}}J\left(\lambda \right)$$

where *κ* ^2^ is the orientation factor (typically assumed to be 2/3 for random orientations), *n* is the refractive index of the medium, Φ_*D*_ is the fluorescence quantum yield of the donor, *N*_A_ is Avogadro’s number, and *J*(λ) is the spectral overlap integral

9 $$J\left(\lambda \right)=\int_{0}^{\infty }F_{D}\left(\lambda \right)\epsilon_{A}\left(\lambda \right)\lambda^{4}d\lambda$$

In this expression, *F*_*D*_(λ) represents the normalized donor emission spectrum and ϵ_*A*_(λ) is the molar extinction coefficient of the acceptor.

For a cascade where the *n*-th protein acts as the donor for the (*n* + 1) -th protein, the rate equation for the acceptor stage becomes

10 $$\frac{dN_{n+1}^{*}\left(t\right)}{dt}=E_{n,n+1}\frac{N_{n}^{*}\left(t\right)}{\tau_{n,\mathrm{eff}}}-\frac{N_{n+1}^{*}\left(t\right)}{\tau_{n+1,\mathrm{eff}}}$$

This formulation replaces radiative coupling with direct dipole–dipole interaction, ensuring that the excitation “flow” is proportional to the donor’s decay rate modulated by the Förster efficiency. The final output power *P*_*f*_(*t*) from the terminal protein in the chain is then

11 $$P_{f}\left(t\right)=\frac{N_{\mathrm{out}}^{*}\left(t\right)\cdot \phi_{f,\mathrm{eff}}}{\tau_{f,\mathrm{eff}}}\cdot E_{\mathrm{photon}}$$

### Intergate FRET-STED Coupling

To achieve cascaded logic, the output of one gate is coupled to the input of the next one via a FRET-STED mechanism. In this configuration, the terminal acceptor of Gate *N* acts as a donor that nonradiatively triggers stimulated emission in the primary fluorophore of Gate *N*+1. The rate of stimulated depletion Γ_STED_ in the second gate is proportional to the excited state population of the previous gate’s output protein *N*_out, *G*1_^*^

12 $$\Gamma_{\mathrm{STED}}=\frac{1}{\tau_{\mathrm{out},G1}}\cdot E\cdot N_{\mathrm{out},G1}^{*}\left(t\right)$$

The modified rate equation for the first protein in the second gate (*N*_1, *G*2_^*^) becomes

13 $$\frac{dN_{1,G2}^{*}\left(t\right)}{dt}=\sigma_{\mathrm{exc}}I_{e}\left(N_{\mathrm{tot}}-N_{1,G2}^{*}\right)-\left(\Gamma_{\mathrm{STED}}+\frac{1}{\tau_{1,\mathrm{eff}}}\right)N_{1,G2}^{*}$$

### Orientation and Geometric Constraints

The FRET efficiency is highly sensitive to the relative orientation of the donor and acceptor transition dipoles, characterized by the orientation factor *κ* ^2^. In this model, the effective Förster radius *R*_0_ is scaled relative to the standard isotropic value *R*_0,avg_ (where *κ* ^2^ = 2/3)

14 $$R_{0}=R_{0,\mathrm{avg}}\cdot {\left(\frac{3\kappa^{2}}{2}\right)}^{1/6}$$

where *κ* ^2^ is defined by the geometric relationship between the dipoles

15 $$\kappa^{2}={\left(\cos\theta_{T}-3\cos\theta_{D}\;\cos\theta_{A}\right)}^{2}$$

For fixed configurations, *κ* ^2^ can range from 0 to 4.

### Substrate-Induced Quenching and Lifetime Modification

When the protein arrangement is immobilized on a metal substrate (e.g., gold), the proximity to the metal surface introduces nonradiative decay pathways through surface energy transfer (SET). This interaction modifies the intrinsic fluorescence lifetime τ_*f*_. Following the nanometal surface energy transfer (NSET) model^18^ for dipole-surface interactions, the effective fluorescence lifetime τ_*f*,*eff*_ as a function of the distance *d*_sub_ from the substrate is given by

16 $$\tau_{f,\mathrm{eff}}\left(d_{\mathrm{sub}}\right)=\frac{\tau_{f}}{1+{\left(\frac{d_{0}}{d_{\mathrm{sub}}}\right)}^{3}}$$

where τ_*f*_ is the intrinsic lifetime in a vacuum or buffer, and *d*_0_ is the critical quenching distance (NSET radius) at which the quenching efficiency is 50%. For a bulk gold substrate, the exponent *n* = 3 corresponds to the point-to-surface energy transfer mechanism.

The modification of the lifetime directly impacts the quantum yield ϕ_*f*,eff_, which is scaled proportionally

17 $$\phi_{f,\mathrm{eff}}\left(d_{\mathrm{sub}}\right)=\phi_{f}\frac{\tau_{f,\mathrm{eff}}\left(d_{\mathrm{sub}}\right)}{\tau_{f}}$$

This refinement allows for a quantitative assessment of the signal attenuation and fast reset times caused by plasmonic quenching, ensuring the model accounts for the physical reality of the supporting architecture.

The reduction in the fluorescence lifetime also influences the dipole–dipole coupling efficiency. Since the Förster radius *R*_0_ is proportional to the sixth root of the donor’s quantum yield (ϕ_*D*_), the proximity to the metal substrate effectively shrinks the interaction range. The substrate-modified Förster radius *R*_0,eff_ is defined as

18 $$R_{0,\mathrm{eff}}=R_{0,\mathrm{orig}}\cdot {\left(\frac{\phi_{f,\mathrm{eff}}\left(d_{\mathrm{sub}}\right)}{\phi_{f}}\right)}^{1/6}=R_{0,\mathrm{orig}}\cdot {\left(\frac{1}{1+{\left(\frac{d_{0}}{d_{\mathrm{sub}}}\right)}^{3}}\right)}^{1/6}$$

Consequently, the FRET efficiency *E* must be recalculated using this effective radius to account for the diminished transfer capability in quenched environments

19 $$E_{\mathrm{eff}}=\frac{1}{1+{\left(d/R_{0,\mathrm{eff}}\right)}^{6}}$$

By incorporating these dependencies, the model accurately reflects how the substrate’s quenching effect simultaneously accelerates the gate reset time while necessitating a closer donor–acceptor spacing to maintain robust logic levels.

### Thermal Stability and Energy Budget

To address potential photothermal denaturation, we evaluate the system’s steady-state temperature rise under operational intensities. Rather than assuming the cluster area acts as a bulk absorber, we model the total heat power generated, *Q*_gen_, as the sum of individual molecular absorption events

20 $$Q_{\mathrm{gen}}=N\cdot \left(\sigma_{\mathrm{exc}}I_{\mathrm{exc}}+\sigma_{\mathrm{sted}}I_{\mathrm{sted}}\right)$$

where *N* is the number of proteins in the aggregate, and σ_exc_ and σ_sted_ are the respective absorption cross sections.

The steady-state temperature increase Δ*T* at the protein–substrate interface is determined by the balance between this localized heat generation and conduction into the surrounding media. Using the characteristic length of the cluster, $\sqrt{A_{\mathrm{cluster}}}$ , the thermal shift is modeled as

21 $$\Delta T=\frac{Q_{\mathrm{gen}}}{\chi \cdot \left(\kappa_{\mathrm{buffer}}+\kappa_{\mathrm{sub}}\right)\cdot \sqrt{A_{\mathrm{cluster}}}}$$

where *κ*_buffer_ and *κ*_sub_ are the thermal conductivities of the medium and substrate, respectively, and χ is a geometric scaling factor (typically χ ≈ 2 for a disk-shaped source on an infinite half-space). This refinement accounts for the semitransparent nature of the protein cluster while maintaining a conservative estimate for heat dissipation limits.

## Results and Discussion

### Arrangement

The concept is demonstrated through simulations of a NOR gate arrangement using readily available protein building blocks. The gate is designed around Enhanced Green Fluorescent Protein (EGFP), mOrange2, and mKate2. In this arrangement, EGFP functions as the NOR gate (referred to as “*protein*_1_”), while mOrange2 and mKate2 serve as the coupling proteins (referred to as “*protein*_2_” and “*protein*_3_”, respectively). The molecules are placed in close proximity to one another, as shown in Figure 2. All relevant properties of these molecules needed for the simulations are provided in Table 1. The data were sourced from.^19–28^

**Figure 2:**
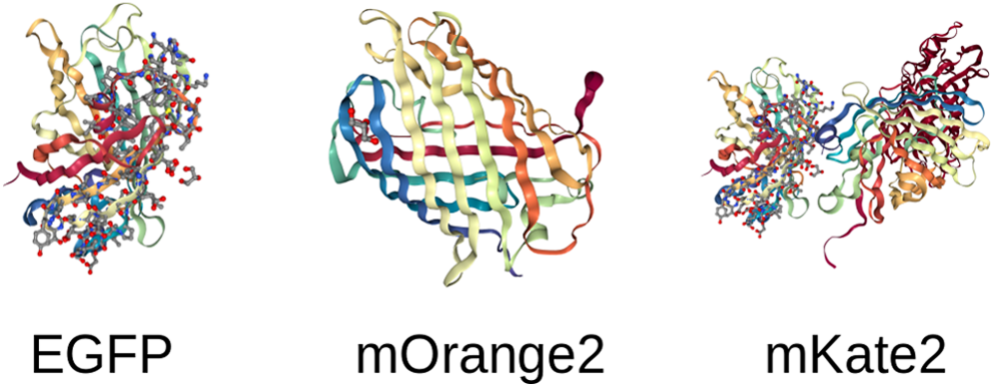
EGFP – mOrange2 – mKate2 NOR gate arrangement. The images of the protein structures were displayed using NGL Viewer, the structure files were obtained from the Protein Data Bank (PDB).

**Table 1:** Parameters of the Proteins in the NOR Gate Arrangement

	EGFP	mOrange2	mKate2
excitation wavelength	488 nm	549 nm	588 nm
excitation spectrum	450–510 nm	500–560 nm	550–600 nm
emission wavelength	509 nm	565 nm	633 nm
fluorescence quantum yield	0.6	0.6	0.4
fluorescence lifetime	2.71 ns	2.8 ns	2.5 ns
peak extinction coefficient	56000 M^–1^cm^–1^	58000 M^–1^cm^–1^	62500 M^–1^cm^–1^
absorption cross-section	2.1 × 10^–16^ cm^2^	2.2 × 10^–16^ cm^2^	2.4 × 10^–16^ cm^2^
STED cross-section	1 × 10^–17^ cm^2^	-	-
suitable STED wavelength	592–640 nm	-	-

The frequency-dependent absorption cross sections σ­(ω) were derived from the molar extinction coefficients ϵ­(λ) provided in the literature for EGFP, mOrange2, and mKate2. The relationship is governed by the expression

22 $$\sigma \left(\lambda \right)=\frac{1000\cdot \ln\left(10\right)}{N_{A}}\epsilon \left(\lambda \right)\approx 3.82\times 10^{-21}\cdot \epsilon \left(\lambda \right)\left[{\mathrm{cm}}^{2}\right]$$

where *N*_A_ is the Avogadro constant. This conversion allows for the direct determination of the transition rates within the dynamic model, ensuring that the simulated intensities for excitation and STED remain within the physical bounds defined by the molecular properties of the fluorescent proteins.

The system operates as follows: EGFP (*protein*_1_) is continuously excited at 488 nm, which causes it to emit fluorescent radiation at 509 nm. This emission is then used to excite mOrange2 (*protein*_2_), which subsequently outputs photons with a 565 nm wavelength. This radiation is sufficient to excite the final molecule in the arrangement, mKate2 (*protein*_3_), which then emits 633 nm radiation; this 633 nm emission corresponds to a logic “1” output.

To achieve a logic “0”, a 633 nm beam, corresponding to input logic “1”, is applied simultaneously with the 488 nm excitation beam. This STED beam effectively depopulates the excited state of EGFP. This results in the cessation of its fluorescence, thereby preventing the cascade that produces the 633 nm output and yielding a logic “0”. This setup thus functions as a NOR gate, which can be coupled to another identical gate.

### Simulation Setup

Simulations were performed in Pydroid 3 using a custom Python numerical solver based on the derived rate equations. To simplify the computational model, the band-pass filtering characteristics of the individual radiations were omitted. We assumed a unidirectional excitation chain where the radiant power from *protein*_1_ excites *protein*_2_, and *protein*_2_ excites *protein*_3_, without back-propagation of radiation. This simplification is physically justified because the fluorescent emission of a given protein in the cascade is red-shifted well beyond the excitation spectrum of its preceding donor.

The baseline simulation environment was defined by several parameters to ensure a realistic assessment of the gate’s performance. Specifically, for the molecular density, each protein cluster consists of *N* = 100 molecules. Furthermore, the intermolecular spacing—defined as the lateral distance between adjacent protein aggregates—is set to *d* = 5 nm to ensure efficient FRET-based coupling. In our simulations, we utilized isotropic Förster distances of *R*_0,12_ = 5.4 nm for the EGFP–mOrange2 transition and *R*_0,23_ = 5.9 nm for the mOrange2–mKate2 transition based on the FPbase FRET Calculator tool.^28^ In the absence of empirical STED-FRET data for the mKate2-EGFP pair, we used the Förster radius of *R*_0,31_ = *R*_12_ = 5.4, which is a standard value, and thereby we utilized *E*_31_ ≈ *E*_12_ as a proxi for the energy transfer.

Regarding substrate proximity, we assume a gold substrate with a quenching distance of *d*_0_ = 4.4 nm.^18^ The protein clusters are positioned at a distance of *d*_sub_ = 10 nm from the substrate, an arrangement where the NSET-induced reduction in fluorescence lifetime is minimal, thereby allowing for an evaluation of the gate’s intrinsic temporal dynamics. Additionally, the orientation factor of the transition dipoles, *κ* ^2^, is set to unity, which represents a “pessimistic” approach. Finally, the continuous fluorescence excitation for *protein*_1_ (EGFP) is maintained at an excitation irradiance of *I*_EXC_ = 10^5^ W/cm^2^.

### Transient Pulse Response and Cascade Signal Integrity

To evaluate the operational limits of the photon-coupled NOR gate, we simulated the system’s response to STED pulses of 10 ns, 1 ns, and 1 ps durations at 10^8^ W/cm^2^, 10^9^ W/cm^2^ and 10^11^ W/cm^2^ irradiance levels, respectively (see Figures 3,4,5). A shorter pulse requires a higher irradiance level to achieve a similar magnitude of output response. The temporal evolution of the excited state populations was modeled across a dual-gate cascade to assess both switching speed and signal degradation.

**Figure 3:**
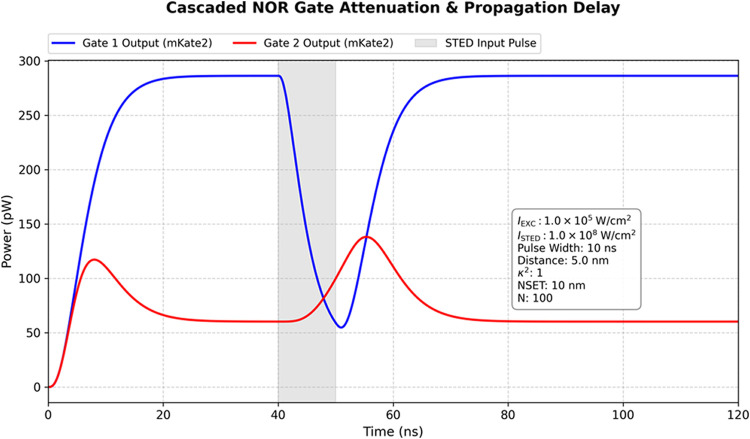
Pulse response with a 10 ns STED pulse, demonstrating the approach to close to steady-state logic levels.

**Figure 4:**
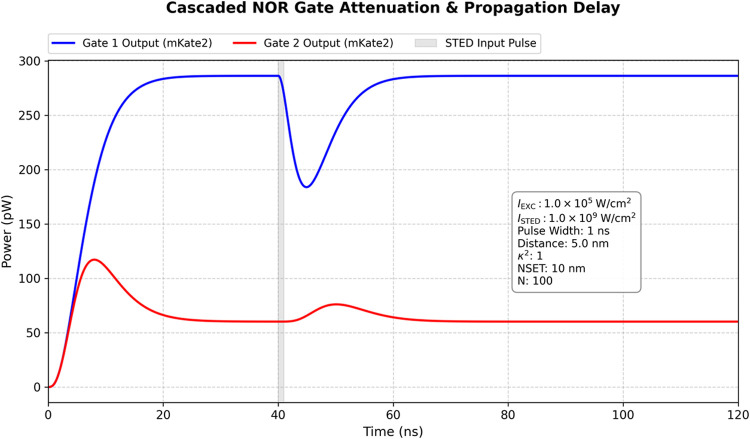
Pulse response with a 1 ns STED pulse, showing moderate signal depth.

**Figure 5:**
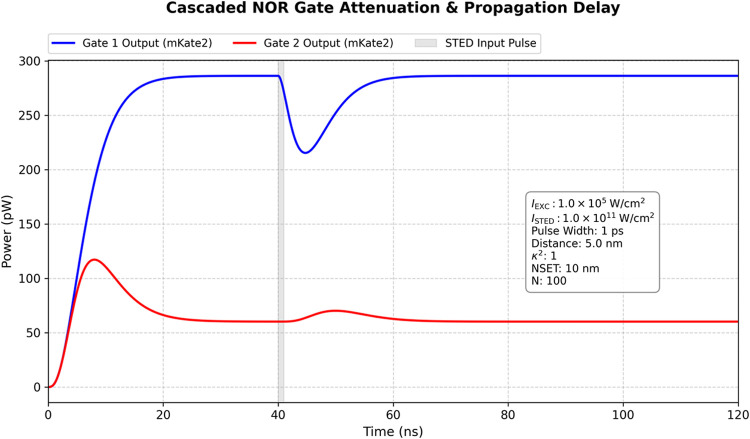
Pulse response of the NOR gate cascade with a 1 ps STED pulse.

#### Temporal Dynamics and the Lifetime Bottleneck

The simulations reveal a significant disparity between the switching mechanism and the signal propagation speed. While the STED-induced depletion of *protein*_1_ (EGFP) occurs on the picosecond scale, the full logic transition is governed by the intrinsic fluorescence lifetimes (τ_*f*_) of the cascade (2.5–2.8 ns).

Several key phenomena define the transient dynamics and operational limits of the gate. First, regarding the relationship between pulse width and contrast, a 1 ps STED pulse is sufficient to trigger a transient dip in the output power, which indicates THz switching capability; however, the STED irradiance level required to achieve a reasonable contrast between the logic “0” and logic “1” levels is 3 orders of magnitude higher than in the case of the 10 ns pulse. Second, examining the relaxation tails reveals that after the STED pulse ends, the system exhibits a nanosecond-scale “recovery tail”. This suggests that while individual switching events are ultrafast, the effective clock frequency for a single gate is limited to the sub-GHz range unless the fluorescence lifetimes are further shortened via NSET coupling to the metal substrate. Finally, signal attenuation poses an additional challenge in a serial cascade of two gates, where we observe a cumulative decrease in the logic “1” level and the contrast between the two logic levels. The lack of intrinsic gain in the photon-coupling mechanism leads to a narrowing of the logic window, emphasizing the need for periodic amplification. For an input pulse of 10 ns, the contrast between the two logic levels at the output roughly halves when a cascade of two gates are used instead of one, which permits the application of several gates before an amplifying element is needed. However, as the duration of the input pulse is shortened, the signal degradation in the two-gate system becomes more prominent. Specifically, when the input is of 1 ps duration, the contrast degradation between the two logic levels is much more significant.

#### Frequency Response and Intersymbol Interference (ISI)

To determine the maximum operational clock speed of the photon-coupled logic, we simulated the system’s response to a continuous 101010 bitstream at two distinct frequencies: 10 and 100 MHz (see Figures 6,7). The simulations utilized an excitation irradiance (*I*_EXC_) of 10^5^ W/cm^2^ and a STED irradiance (*I*_STED_) of 10^9^ W/cm^2^.

**Figure 6:**
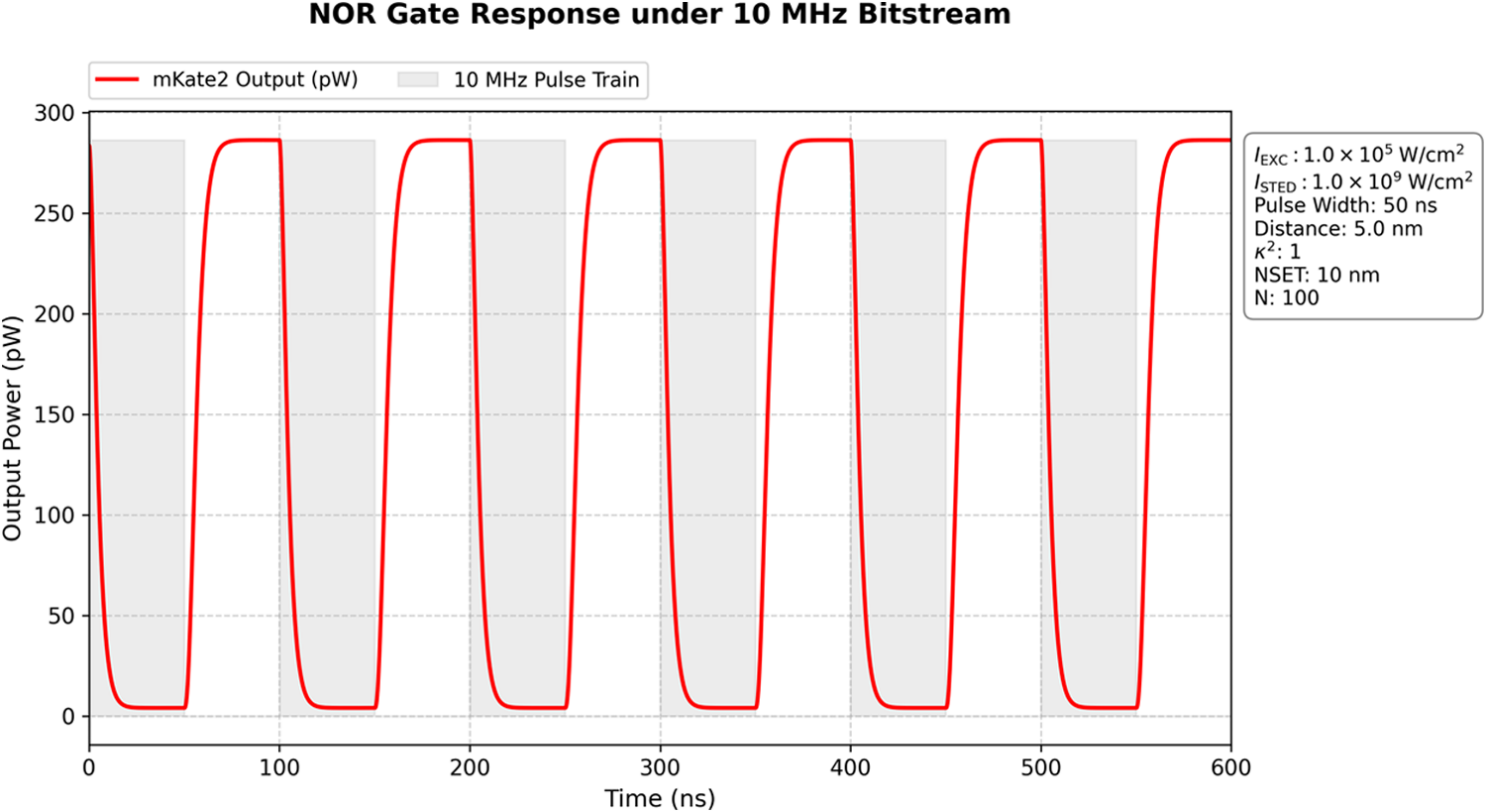
Ten MHz pulse train response. The 100 ns bit period allows for complete relaxation to logic “1” levels between STED pulses.

**Figure 7:**
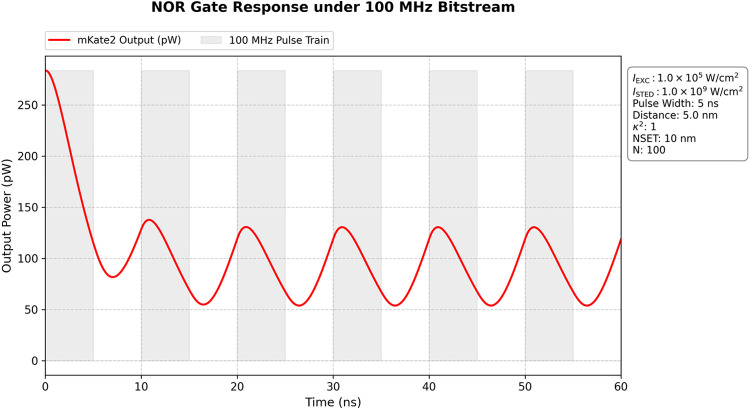
100 MHz pulse train response. Significant intersymbol interference is observed as the 10 ns bit period approaches the cumulative fluorescence lifetime of the cascade.

As shown in Figure 6, at a frequency of 10 MHz, the gate exhibits near-ideal behavior. The 100 ns bit period provides sufficient time for the excited state populations of EGFP, mOrange2, and mKate2 to fully recover to their steady-state values between pulses, resulting in a robust logic “1” level of approximately 300 pW and a high contrast ratio.

In contrast, the 100 MHz simulation (Figure 7) reveals the onset of significant intersymbol interference (ISI). At this frequency, the 10 ns bit period is comparable to the relaxation ”tails” of the cascaded proteins. Consequently, the output power fails to return to the baseline logic “1” level before the arrival of the subsequent STED pulse. This leads to a compression of the dynamic range, where the output oscillates between approximately 50 pW (logic “0”) and 135 pW (logic “1”).

The results indicate that the cumulative delay of a three-protein cascade imposes a practical limit on the bandwidth. For the current configuration, 100 MHz represents an upper bound where the narrowing of the logic window would likely necessitate active signal regeneration or more complex thresholding in subsequent stages. The bandwidth can be increased via NSET coupling to the metal substrate.

#### Logic State Definition and Signal Discrimination

The operational distinction between logic “0” and “1” states in the proposed FRET-STED architecture is fundamentally defined by the relative modulation of fluorescence intensity rather than by fixed power thresholds. In a nanoscale, high-speed computing context—particularly in the GHz regime—logic states exist in a photon-starved environment where signal discrimination becomes inherently stochastic.

Consequently, we define the logic window not as a static irradiance level, but as a statistical separation between the unperturbed emission of the donor–acceptor complex (Logic “1”) and the STED-suppressed state (Logic “0”). The resolvability of these states is a function of the integration time (pixel dwell time) and the specific sensitivity characteristics, such as the Dark Count Rate (DCR), of the detection hardware. By maintaining a clear modulation depth, the architecture remains compatible with modern statistical estimation frameworks. Techniques such as Maximum Likelihood Estimation (MLE)^29^ or deep-learning-assisted signal restoration^30^ are capable of robustly reconstructing digital states from sparse photon arrivals, ensuring that the compressed logic windows identified in our simulations are technically viable for high-speed information processing.

#### Thermal Stability Analysis

A critical concern for the physical realization of this system is the potential for photothermal denaturation of the protein clusters. We calculated the steady-state temperature rise (Δ*T*) for a cluster of *N* = 100 proteins under the STED irradiance levels (*I*_*s*_) applied in the simulations, assuming continuous irradiation (see Table 2).

**Table 2:** Calculated Thermal Steady-State Temperature Rise for Varying STED Intensities

STED irradiance (*I*_*s*_)	temperature rise Δ*T* (Ex+STED)
10^8^ W/cm^2^	1.607 × 10^–3^ K
10^9^ W/cm^2^	1.577 × 10^–2^ K
10^11^ W/cm^2^	1.574 × 10 ° K

The results indicate that even at the highest irradiance of 10^11^ W/cm^2^, the temperature rise remains below 1.6 K. The integrated energy delivery is well within the safety margins for biological fluorophores, which typically tolerate temperatures up to 313–323 K before unfolding.

#### Molecular Optimization and Future Prospects

The current performance represents a baseline using nonoptimized, commercially available proteins (EGFP, mOrange2, and mKate2). To reach the theoretical THz limits of STED-based computing, future designs must focus on synthetic chromoproteins with picosecond intrinsic lifetimes^27^ and higher STED cross sections to minimize the recovery bottleneck and improve signal-to-noise ratios in multigate cascades.

### Bandwidth Enhancement via NSET-Induced Lifetime Quenching

To address the ”lifetime bottleneck” observed in the baseline simulations, we investigated the effect of Nanometal Surface Energy Transfer (NSET) by reducing the protein-to-substrate distance (*d*_sub_) to 2 nm. As defined in the model equations, the proximity to a substrate introduces nonradiative decay pathways that scale with 1/*d*_sub_^3^, significantly accelerating the relaxation of the excited state populations at the cost of reduced quantum yield.

#### 1 GHz Operational Performance

The simulation of a 1 GHz pulse train (500 ps pulse width) with *d*_sub_ = 2 nm, *I*_EXC_ = 10^8^ W/cm^2^ and *I*_STED_ = 10^10^ W/cm^2^ reveals a transformative improvement in temporal resolution (see Figure 8).

**Figure 8:**
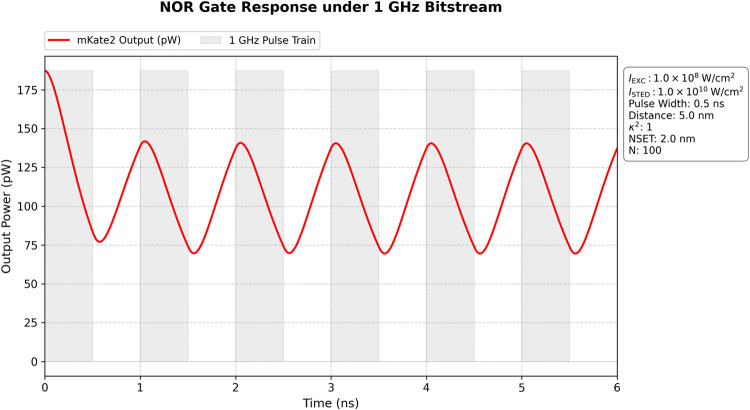
NSET-enhanced 1 GHz pulse train response.

The simulation of a 1 GHz pulse train under these conditions highlights several critical performance dynamics. First, with respect to ultrafast recovery, the effective lifetimes (τ_*f*,eff_) are sufficiently shortened to allow the three-protein cascade to return to a baseline state within the 1 ns bit period. This effectively mitigates the intersymbol interference (ISI) that characterized the unquenched 100 MHz simulations. However, an inherent logic level trade-off emerges, as the enhanced temporal resolution necessitates a compromise in signal amplitude. Consequently, the peak logic “1” power is reduced to approximately 140 pW, compared to the 300 pW observed at a 10 nm substrate distance despite the increased irradiance levels. Nonetheless, the switching contrast remains highly favorable; despite the reduction in absolute power, the contrast ratio remains high. The logic “0” level is suppressed to ∼75 pW, thereby maintaining a clear logic window of approximately 75 pW at gigahertz frequencies.

These results suggest that the operational bandwidth of FRET-coupled molecular logic is a tunable parameter. By engineering the plasmonic environment via substrate proximity, the system can be transitioned from a high-signal/low-speed regime to a lower-signal/high-speed (GHz) regime.

### Impact of Dipole Orientation on Cascade Signal Integrity

The geometric orientation of transition dipoles, characterized by the orientation factor *κ* ^2^, is a critical parameter in the Förster resonance energy transfer (FRET) rate equation. At first glance, we would assume that maximizing *κ* ^2^ (e.g., via site-specific protein immobilization on DNA origami) would significantly improve signal-to-noise ratios. However, our multistage cascade simulations reveal a more nuanced behavior (Figure 9).

**Figure 9:**
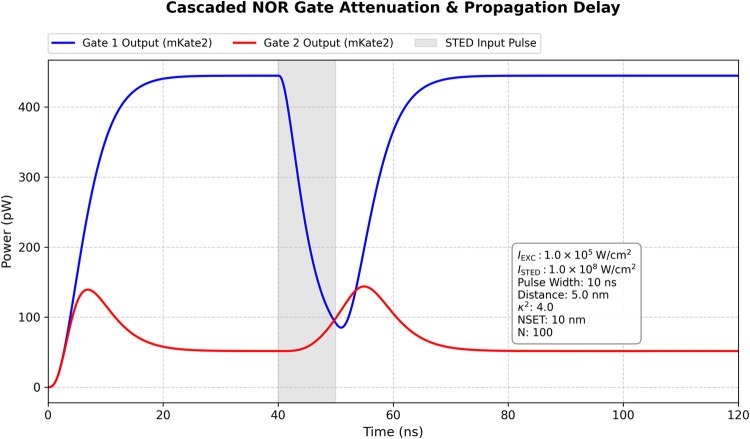
Pulse response with a 10 ns STED pulse in case of optimal dipole–dipole orientation.

While an increase in *κ* ^2^ from 1.0 (random orientation) to 4.0 (perfectly aligned) yields a significant, more than 50% increase in the peak output power of the primary gate (*P*_out,1_), this gain is substantially attenuated in subsequent stages. For the secondary gate (*P*_out,2_), the same 4-fold increase in *κ* ^2^ results in a significantly less pronounced improvement in logic “1” signal levels.

This observation can be attributed to two competing physical effects inherent to the FRET-STED NOR gate architecture. On one hand, the system experiences (1) internal gain enhancement, where improved dipole alignment increases the internal energy transfer efficiency within a single gate, thereby maximizing the energy transfer toward the terminal emitter. On the other hand, this is balanced by (2) inhibitory coupling feedback; because the logic “1” output of Gate *n* serves as the depletion trigger (STED pulse) for Gate *n* + 1, any gain in the primary gate’s emission power results in a concomitantly stronger inhibitory signal sent to the next stage.

These results suggest a significant design advantage: the FRET-STED NOR gate demonstrates a degree of geometric stability. The architecture is remarkably robust against angular jitter and suboptimal protein orientation, maintaining functional logic levels across a wide range of *κ* ^2^ values. This robustness reduces the stringency of manufacturing tolerances.

### Sensitivity to Intermolecular Spacing

The physical feasibility of the photon-coupled logic gate relies on the precise spatial arrangement of the protein clusters. Given the 1/*d* ^6^ dependency of the Förster resonance energy transfer (FRET) mechanism (eq 7), small variations in the intermolecular distance *d* significantly impact the signal integrity of the cascade. We performed a sensitivity analysis by varying *d* between 4 and 6 nm.

#### Distance-Dependent Signal Attenuation

The simulation results (Figures 10 and 11) highlight the dependence of signal attenuation on the intercluster distances of the logic gates. On one hand, regarding coupling degradation at 6 nm, increasing the distance to this value results in a noticeable loss of signal in the second gate, causing the peak power of Gate 2’s output to drop to ∼100 pW. Conversely, enhancing the performance by reducing the distance to 4 nm yields a robust Gate 2 response, with peak power levels reaching ∼150 pW. Ultimately, evaluating these variations outlines the tolerance constraints of the system, suggesting that a 1–2 nm error in molecular placement is still tolerable by the arrangement.

**Figure 10:**
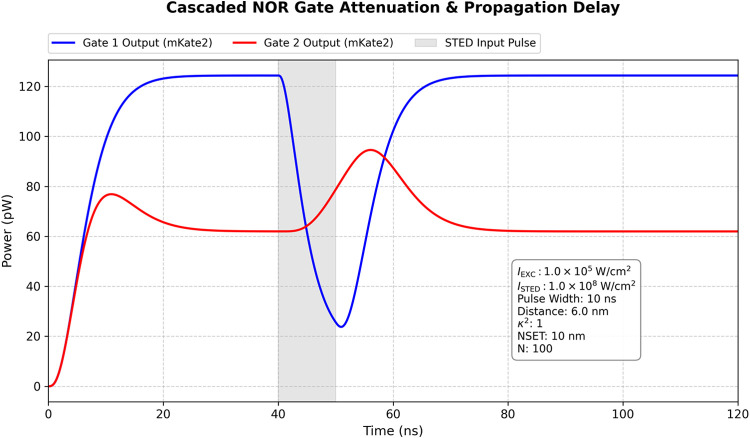
Pulse response with a 10 ns STED pulse in case of a 6 nm intermolecular distance.

**Figure 11:**
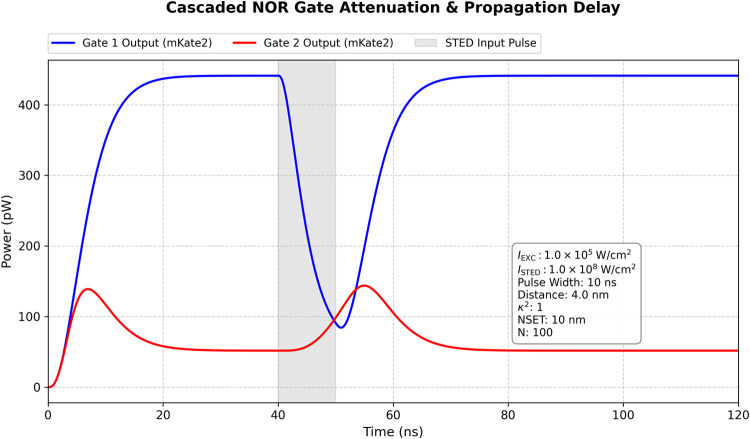
Pulse response with a 10 ns STED pulse in case of a 4 nm intermolecular distance.

### Impact of Molecular Cluster Size on Gate Performance and Robustness

The transition from single-molecule logic to protein clusters (*N* ≈ 100) introduces a critical design trade-off between signal integrity and thermal management. We identify three primary advantages to the ensemble approach. First, the signal-to-noise ratio (SNR) and logic contrast are significantly enhanced; while the logic swing increases linearly with *N*, the stochastic shot noise scales only as √*N*. This widening of the noise margin is essential for maintaining bit integrity in multigate cascades.

Second, the cluster architecture provides inherent resistance to manufacturing and environmental variances. In a single-molecule pair, subnanometer fluctuations in distance or unfavorable transition dipole orientations (*κ* ^2^) can lead to total gate failure. In a cluster, these parameters are averaged across the ensemble. Following the Law of Large Numbers, the aggregate transfer function converges to the nominal theoretical model, effectively “smoothing out” the impact of nanoscale positioning jitters. Third, the system exhibits functional redundancy; the logic gate remains operational even if a fraction of the molecules undergo irreversible photobleaching or denaturation.

While ensemble clustering successfully averages out nanoscale positioning jitters, it may introduce minor inhomogeneous broadening due to local substrate microenvironments. However, the broad spectral overlap integrals inherent to the selected fluorescent proteins mitigate this effect. To eliminate unintended intergate cross-talk (spurious FRET) in dense circuits, DNA origami can be exploited as a three-dimensional breadboard to enforce spatial isolation—such as arranging adjacent logic paths on opposite faces of a 3D origami block—or by utilizing spectrally orthogonal protein cascades for parallel channels.

However, these benefits necessitate a careful assessment of the thermal budget. The steady-state temperature rise Δ*T* is governed by the total heat power *Q*_gen_ and the effective dissipation area *A*. While *Q*_gen_ scales linearly with *N* due to additive molecular absorption, the dissipation area also expands proportionally (*A* ∝*N*). Given that Δ*T* is inversely proportional to the characteristic dimension of the heat source (√*A*), the resulting temperature rise follows a square-root scaling law

23 $$\Delta T\propto \frac{Q_{\mathrm{gen}}}{\sqrt{A}}\propto \frac{N}{\sqrt{N}}=\sqrt{N}$$

Consequently, a cluster of *N* = 100 proteins experiences a 10-fold increase in local heating compared to a single-molecule gate. Nevertheless, since the total energy dissipation remains small and the gate’s footprint is orders of magnitude smaller than the thermal diffusion length of the substrate, the absolute temperature rise remains well below the thermal denaturation threshold of fluorescent proteins even if *N* is increased well above 100.

### Photostability, Operational Lifetime, and Mitigation Strategies

A fundamental constraint in all-optical protein logic is the irreversible photodamage of the fluorophores. The operational durability of the proposed NOR gate is governed by the photobleaching quantum yield (Φ_*b*_), which represents the probability of a destructive photochemical reaction occurring per excitation event.

#### Theoretical Dependencies

The operational lifetime of a single gate (τ_gate_) can be expressed as a function of the switching frequency (*f*), the molecular population density (*N*), and the required photon flux for signal coupling (*n*_ph_)­

24 $$\tau_{\mathrm{gate}}\propto \frac{N}{\Phi_{b}\cdot f\cdot n_{\mathrm{ph}}}$$

This relationship highlights that while THz-scale switching is theoretically supported by the STED transition, the “photon budget” of a static molecular ensemble is finite. As *f* increases, the residency time of the protein in the functional state decreases unless *N* is scaled or restorative mechanisms are employed to maintain the population.

#### Engineering and Environmental Solutions

To address these limitations and satisfy the requirements for a practical computing substrate, we propose a three-tiered mitigation strategy. First, our approach involves (1) the genetic engineering of ultrastable scaffolds, noting that the implementation of the gate is not restricted to traditional fluorescent proteins. The use of recently evolved ultraphotostable variants, such as *StayGold*, provides an order-of-magnitude increase in τ_*gate*_ due to their significantly reduced Φ_*b*_ and enhanced structural rigidity, which limits oxygen access to the chromophore.^31^ Second, the strategy relies on (2) differential FRET protection, where we identify a “photoprotective” effect inherent in the FRET-coupled architecture.^32^ Because the FRET pathway provides a rapid nonradiative de-excitation route for donor proteins, it reduces their *S*_1_ excited-state residency time, thereby effectively “hardening” the internal logic nodes against bleaching compared to isolated emitters and shifting the primary burden to the final output clusters. Finally, we propose (3) microfluidic replenishment to achieve indefinite operation, establishing a microfluidic substrate where the protein ensemble is maintained in a steady state.^33^ By implementing an oxygen-scavenged, reducing environment (e.g., using enzymatic scavengers), the triplet-mediated bleaching pathway can be suppressed. Furthermore, a flow velocity (*v*) can be tuned such that the residency time of a protein in the optical voxel (*L*) satisfies *L*/*v* ≪ τ_bleach_, ensuring that bleached molecules are replaced before signal degradation occurs. While integrating fluid flow with high-precision optical addressing poses technical challenges, this may be mitigated by the rigid chemical immobilization of the supporting DNA origami breadboards to the solid substrate. Alternatively, fluid replacement can operate via a periodic or low-velocity regime between computational cycles.

#### Restorative Photorecovery

Beyond irreversible bleaching, we consider the role of reversible “dark states.” Existing literature suggests that specific “reset” wavelengths (e.g., 405 nm) or multiphoton IR pulses can facilitate the recovery of proteins from nonfluorescent isomers back to the functional *cis*-state.^32^ Incorporating a low-intensity maintenance beam into the architecture could further extend the Mean Time Between Failures (MTBF) of the logic substrate by constantly recycling the dark-state population.

### Signal Amplification and RSFP-Based Modulation

A critical challenge in cascaded all-optical logic is the lack of intrinsic signal gain, as FRET transfer efficiencies (η_FRET_) and collection losses inevitably lead to signal degradation. To achieve a fan-out >1, we propose the integration of Reversibly Switchable Fluorescent Proteins (RSFPs) acting as optical amplifiers.

In this architecture, the output of a primary NOR gate serves as a switching signal for an RSFP-based “optical transistor.” While biological RSFPs such as *Dronpa* or *Padron* typically exhibit slow switching kinetics in the millisecond range due to protein backbone rearrangement, the fundamental physical limit is governed by the chromophore’s initial photoisomerization event.

Quantitatively, the *cis–trans* isomerization occurs on a time scale of τ_iso_ ≈200 fs to 1 ps.^8–10^ The observed “slowness” in current systems is a result of the low quantum yield of switching (Φ_sw_) and the steric hindrance provided by the protein’s internal cavity.^34,35^ We posit that by engineering “loose-cage” protein variants or utilizing purely electronic switching mechanisms, such as Photoinduced Electron Transfer (PET),^36^ the modulation speed could approach the vibrational relaxation limit

25 $$f_{\max}\approx \frac{1}{\tau_{\mathrm{vib}}}\approx 0.1-1\mathrm{THz}$$

This suggests that RSFP-integrated protein logic is not fundamentally limited to low-speed applications, but could theoretically support THz-rate signal regeneration, provided that the stochastics of the switching event are mitigated through nanocluster redundancy.

## Experimental Roadmap for Validation

To transition the proposed protein-based logic from a theoretical model to a physical implementation, we outline a two-tiered experimental strategy focusing on nanoscale precision and ensemble verification.

### Nanoscale Assembly via DNA Origami

The primary challenge in realizing the NOR gate architecture is the requirement for sub-10 nm interprotein spacing to ensure efficient FRET coupling. We propose using *DNA origami nanostructures* as a programmable breadboard.^11,37,38^ By functionalizing specific DNA staples with protein-binding ligands (e.g., biotin–streptavidin or Ni-NTA for His-tagged proteins),^39^ donor and acceptor FPs can be positioned with 1–2 nm precision.^40,41^ This approach allows for the creation of rigid, spatially defined logic tiles^11^ that can be deposited on a glass substrate for optical characterization.

### Verification Using STED-TCSPC Microscopy

To validate the switching dynamics of the FRET-STED cascade, we propose an experimental setup utilizing a modified STED microscope integrated with Time-Correlated Single Photon Counting (TCSPC) electronics.^29,30^ Unlike traditional optical logic gates that use excitation pulses as triggers, this architecture treats the depletion beam as the active logic signal. To establish the system’s logic bias via continuous excitation, a single laser source (e.g., 488 nm for EGFP) provides continuous or high-frequency pulsed excitation to the primary gate protein (*protein*_1_), maintaining the system in a baseline “on” state (Logic output “1”). For the logic inputs, two independent or split STED-wavelength beams (e.g., 633 nm) serve as Logic Inputs A and B, which are focused on the gate-protein aggregate using high-numerical aperture optics to ensure localized depletion. The fundamental switching mechanism relies on the fact that the presence of either STED input beam (Input Logic “1”) triggers near-instantaneous electronic depopulation of the donor’s excited state. This effectively “quenches” the EGFP and halts the energy transfer cascade to the terminal mKate2, thereby facilitating the NOR operation.^12^

To evaluate the readout and contrast of the system, the Logic Output is determined by monitoring the fluorescence of the terminal acceptor (mKate2) via a TCSPC module,^29^ where a successful NOR operation is confirmed when the terminal emission is suppressed (Logic “0”) by the presence of either STED input. Finally, regarding signal integrity, Maximum Likelihood Estimation (MLE) or deep-learning-based phasor analysis (e.g., flimGANE) is employed to ensure accuracy in the high-speed, photon-starved regimes inherent to nanoscale logic.^29,30^ These methods successfully distinguish between the quenched (Logic ‘0’) and unquenched (Logic “1”) states based on the donor’s shortened lifetime, providing robust discrimination independent of local concentration fluctuations.

### Ensemble-Based Proof-of-Concept

As a preliminary step, the core nonlinear switching principle can be validated in a bulk environment. A thin-film PVA (Poly­(vinyl alcohol)) matrix doped with a calibrated ratio of Donor–Acceptor FRET pairs can be used to observe the ensemble “quenching” response.^42^ By measuring the change in Donor lifetime as a function of STED intensity applied to the Acceptors, the fundamental transfer function can be verified without the immediate need for complex nanostructure fabrication.

#### Signal Integrity and Contrast Metrics

To ensure robust logic discrimination, we propose a ratiometric or lifetime-based readout rather than simple intensity measurements.^29^ By employing TCSPC to monitor the fluorescence lifetime of the donor protein, the logic state “0” (quenched) can be distinguished from “1” (unquenched) with high fidelity, independent of local protein concentration fluctuations.

## Conclusions

In this work, we have presented a comprehensive theoretical framework and simulation-based validation for an all-optical universal logic architecture based on multistage FRET-STED energy cascades in fluorescent protein nanoclusters. By shifting the logic switching mechanism from slow protein backbone rearrangements to near-instantaneous electronic depletion via Stimulated Emission Depletion (STED), the theoretical feasibility of molecular computing operating at speeds ranging from 1 GHz to 1 THz.

Our analysis reveals several critical insights into the performance and physical constraints of this platform. First, considering baseline performance and the “lifetime bottleneck”, simulations using nonoptimized, commercially available proteins (EGFP, mOrange2, and mKate2) establish a robust baseline for the technology. While the STED-induced switching event occurs on a picosecond scale, the overall logic transition speed in these unoptimized clusters is governed by the intrinsic fluorescence lifetimes (typically 2.5–2.8 ns), which limits the effective clock frequency to the sub-GHz range. Crucially, this “lifetime bottleneck” is not a fundamental limit of the FRET-STED architecture, pointing to the platform’s THz-speed realizability. Because the initial photoisomerization and vibrational energy redistribution occur on a sub-100 fs time scale, which corresponds to a theoretical experimental bandwidth of approximately 3.15 THz, we have shown that focusing on synthetic chromoproteins with picosecond intrinsic lifetimes and higher STED cross sections makes these THz-speed building blocks theoretically realizable, as supported by established electronic transition limits.

Furthermore, we achieved bandwidth enhancement via NSET, demonstrating that the operational bandwidth can be actively tuned by engineering the plasmonic environment. By placing the protein clusters in close proximity (∼2 nm) to a gold substrate, Nanometal Surface Energy Transfer (NSET) effectively shortens the fluorescence lifetimes, enabling robust 1 GHz operation even with current protein variants. Beyond these electronic dynamics, geometric and spatial sensitivity remains paramount, as the integrity of the logic signal is highly sensitive to the nanometer-scale arrangement of the proteins; our analysis confirms that an intermolecular spacing of 4–6 nm is required for efficient FRET coupling. In addition to spatial precision, thermal and operational stability must be maintained. Simulations indicate that the system remains thermally stable even under continuous high-intensity STED irradiance (up to 10^11^ W/cm^2^), with a calculated temperature rise of less than 1.6 K, which is well below the denaturation threshold for fluorescent proteins. To sustain this performance, we have proposed strategies such as using ultrastable variants like *StayGold* and microfluidic replenishment to address photobleaching and maintain a continuous photon budget. Ultimately, this establishes a clear path toward scalability. To overcome the lack of intrinsic signal gain, we have outlined a roadmap for integrating Reversibly Switchable Fluorescent Proteins (RSFPs) as optical amplifiers, which, combined with the use of DNA origami as a molecular breadboard for precise positioning, provides a clear experimental path toward realizing complex, high-speed molecular logic circuits.

This work establishes a novel materials platform that bypasses the kinetic limits of traditional molecular logic, offering a viable pathway toward nanoscale, all-optical computing at THz bandwidths.
